# Veratrum in Puerperal Convulsions

**Published:** 1887-02

**Authors:** A. H. McCord

**Affiliations:** Rusk, Texas


### VERATRUM IN PUERPERAL CONVULSIONS.

*By A. H. McCord, M. D., Rusk, Texas.*

For Daniel's Texas Medical Journal.

HAVING read in the December number of your JOURNAL an article from Dr. U. G. M. Walker on the nse of veratrum viride in the treatment of puerperal eclampsia, and having had a somewhat similar experience in the use of the drug, and believing it to be an excellent auxiliary in the treatment of this dread disease, I wish to add my mite of testimony in its favor, by reporting the following case :

On the morning of May 19th, 1886, was called to see Sallie Mc, negress, aged about 25, multipara, in previous robust health, about 7 months pregnant. When I arrived she had had two convulsions, could yet swallow; was blind. I succeeded in getting her to swallow a large dose of Brom. Pot. Chloral Hydrate, and Sulph. Morph. In one hour she had another convulsion, after which she was no longer able to swallow.

I then unloaded her bowels with an enema. Anticipating another spasm in about one hour, I commenced the the use of chloroform by inhalatiou in time to lengthen the interval to 1¼ hours

The chloroform alone was kept up at intervals, for 3 hours more, with no good result except increasing the interval a little, without mitigating the severity of the spasms. I then bled her freely, still keeping up the chloroform, which lessened the severity of the paroxysms somewhat. Again after 2 hours I reopened the vein and bled her the second time, with no better result than the first.

Just at this juncture I remember to have read a short report on the use of verat. viride in puerperal eclampsia with good results by James L. Sulivan (St. Louis Courier of Medicine, March, '85, page 240), and about 20 minutes before I was expecting the fifteenth convulsion I gave her hypodermically 12 drops Norwood's Tinct. Veratrum Viride with 10 grs. Hydrate Chloral, and the fifteenth paroxysm never came. In 40 minutes I repeated the dose with the happy result that there were no more spasms, neither were there any muscular twitchings, nor even tremor indicating the return of the fits. The great arterial tension was speedily relieved, and the pulse slowed to about 60 beats per minute. I then substituted Tinct. Digitalis for the veratrum, and at intervals of two hours gave the chloral and digitalis until six doses were given. She recovered consciousness in about 20 hours from the last fit.

There was no dilatation of the os nor contraction of the body of the uterus during the whole nervous phenomena, but two days later uterine contractions set up, labor came on and she was soon delivered of a dead fœtus, from all of which she made a speedy recovery.

I report this case in detail to show the comparative depressor motor value of veratrum with the usual remedies exhibited in the treatment of this dread malady, also how promptly the perturbed nervous condition responded to its sedative action.

